# Agentic AI as a dual-role lecturer and teaching assistant: effects on learner autonomy, self-regulated learning, and intrinsic motivation in university-level EFL education

**DOI:** 10.3389/fpsyg.2026.1847607

**Published:** 2026-06-25

**Authors:** Kuei-Hao Li, Chia-Kai Chang

**Affiliations:** 1 International Intercollegiate Ph.D. Program, National Tsing Hua University, Hsinchu, Taiwan; 2 Center for General Education, National Central University, Taoyuan City, Taiwan

**Keywords:** agentic AI, EFL, higher education, learner autonomy, self-regulated learning, socratic dialogue

## Abstract

Large language model (LLM)-powered agentic systems are increasingly deployed in higher education, yet their effects on learner autonomy and self-regulated learning (SRL) remain empirically underexamined. This 16-week quasi-experimental study evaluated a dual-role Agentic AI—an AI Lecturer paired with an adaptive AI Teaching Assistant—embedded in Uedu.tw, a university EFL platform in Taiwan, drawing on Self-Determination Theory (SDT) and Vygotsky's Zone of Proximal Development. Participants were 120 Taiwanese B1 undergraduates (60 experimental, 60 control) assigned by intact class with a single instructor and identical curriculum. ANCOVA showed greater experimental-group gains in learner autonomy (partial η^2^ = 0.247), SRL strategies (partial η^2^ = 0.215), and intrinsic L2 motivation (partial η^2^ = 0.183), with no parallel effect on extrinsic goal orientation—a dissociation consistent with SDT. Because the experimental condition combined AI access with a Socratic-dialogue task format, these effects index the AI-supported condition as a whole and cannot be attributed to the AI system in isolation. Learner-level analysis of 6,235 AI Teaching Assistant queries showed that 54 of 60 experimental learners (90.0%) shifted monotonically from answer-seeking toward metacognitive and elaboration-seeking queries, which we treat descriptively rather than as evidence of internalized self-regulation. Reflexive thematic analysis of 16 interviews yielded three illustrative themes. The findings inform agentic-AI design in Confucian-heritage EFL contexts and underscore the need to distinguish effects with agentic technology from effects of it; whether within-intervention gains persist after scaffolding is withdrawn remains open.

## 1 Introduction

Large language model (LLM)-powered agentic AI systems are reconfiguring the affordances available for second language (L2) pedagogy (Chu et al., 2024). Unlike reactive chatbots, agentic systems are proactive, goal-directed, and capable of dynamically adapting instructional strategies in response to real-time learner performance (Papi and Hiver, 2025). Whether they support the development of learner autonomy—a construct foundational to sustained L2 development (Benson, 2013; Holec, 1981)—or inadvertently substitute for it remains an open question with direct implications for pedagogical design.

Two complementary frameworks anchor this question. Self-Determination Theory (SDT; Deci and Ryan, 2000) identifies autonomy, competence, and relatedness as motivational preconditions for self-directed engagement. Vygotsky's Zone of Proximal Development (ZPD; Vygotsky, 1978) specifies the interactional mechanism: scaffolding pitched just beyond independent capacity, with progressive transfer of regulatory responsibility. Together they imply a stringent design criterion: educational AI should support autonomy needs while actively fading its own scaffolding. Yet AI engineered for accessibility and responsiveness may simultaneously reduce the productive cognitive struggle through which self-regulation is internalized (Chiu, 2024; Darvishi et al., 2024).

Although Self-Determination Theory has long informed motivation research in technology-mediated learning (Ryan and Deci, 2020), its translation into design principles for LLM-powered agentic systems is still emerging. Recent work on AI in higher education has begun to examine autonomy-supportive design choices in conversational AI tutors (Peláez-Sánchez et al., 2024), with mixed evidence on whether these systems promote independent learning or foster dependence (Wu et al., 2026). Parallel work grounded in Vygotsky's Zone of Proximal Development has examined how adaptive scaffolding in intelligent tutoring systems can support gradual release of responsibility (Murray and Arroyo, 2002), though comparable evidence for LLM-based agentic tutors in EFL contexts remains scarce.

Three gaps motivate the present study. Empirically, most AI-assisted L2 work has examined single-function tools rather than dual-role agentic designs in which the system concurrently operates as an instructional agent and a responsive scaffolding partner. Theoretically, although SDT and ZPD are frequently invoked, few studies operationalise both concurrently or provide empirical evidence that scaffolding fading—the mechanism at the heart of ZPD—can be realized in LLM-driven environments. Contextually, Taiwanese higher education EFL exemplifies a Confucian-heritage tradition in which performance-oriented norms coexist with autonomy-supportive aims (Yang, 2024); whether agentic AI can support autonomous learning behaviors in such contexts has not been empirically established.

The present 16-week quasi-experimental study addresses these gaps through a dual-role Agentic AI deployed on Uedu.tw, an open-access university-level EFL platform in Taiwan. Within the Uedu.tw implementation, this system operates through two concurrently active roles: the AI Lecturer (structured input plus Socratic questioning) and the AI Teaching Assistant (response to learner queries via progressive hint scaffolding); a Socratic guardrail at the prompt-architecture level prevents direct answer provision. Two research questions guided the study:

**RQ1**. Does integration of a dual-role Agentic AI on Uedu.tw improve learner autonomy, self-regulated learning strategies, and intrinsic L2 motivation among Taiwanese college EFL students, compared with conventional online instruction?

**RQ2**. How do students perceive the dual-role Agentic AI's pedagogical value, and what themes characterize their experience of AI-mediated scaffolding?

## 2 Method

### 2.1 Participants and design

A quasi-experimental pretest–posttest non-equivalent control group design was employed over a 16-week academic semester. An a priori power analysis (G^*^Power 3.1; Faul et al., 2007) indicated a minimum total *N* = 128 for between-group comparison at α = 0.05, power = 0.80, medium effect (*f* = 0.25; Cohen, 1988). The achieved *N* = 120 fell slightly short of this target [see Section 4.4(4)].

Participants were 120 undergraduates (*M*_*ame*_ = 19.7, *SD* = 1.3; 68 female, 52 male) enrolled in a required general English course at a comprehensive university in northern Taiwan. All were Mandarin-dominant bilinguals; inclusion required B1 CEFR proficiency as verified through the institution's standardized placement examination at matriculation. Group assignment followed intact-class allocation: two parallel sections of the same course were designated experimental (EG; *n* = 60) and control (CG; *n* = 60). Both sections were taught by the same instructor and used identical curriculum materials, weekly task schedules, and assessment criteria. Baseline equivalence on all dependent variables and on prior English learning experience and self-reported technology familiarity was confirmed through independent-samples *t*-tests (all *p* > 0.15). Cluster-level effects arising from intact-class allocation are addressed in Section 4.4.

Data were collected during the Fall 2025 semester (September 2025–January 2026) under the consent and ethics-authorization framework detailed in the Ethics Statement (NTU-REC Approval No. 202507EM058; expedited review).

### 2.2 The Uedu.tw agentic AI system

**Figure 1:**
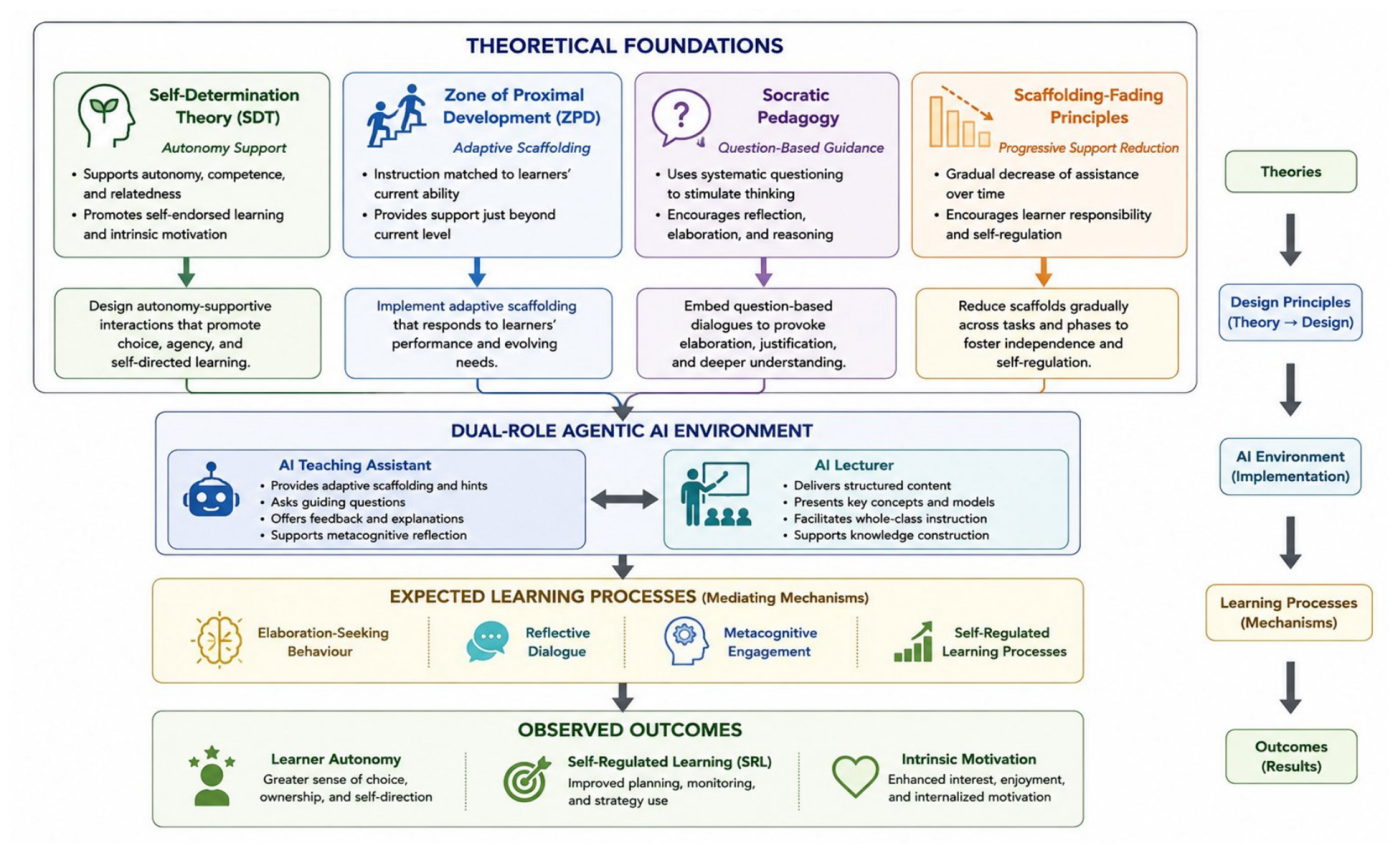
Conceptual framework illustrating how Self-Determination Theory, the Zone of Proximal Development, Socratic pedagogy, and scaffolding-fading principles informed the design of the dual-role agentic AI environment and the hypothesised pathways leading to learner autonomy, self-regulated learning, and intrinsic motivation.

The design of the dual-role Agentic AI environment was directly informed by the theoretical perspectives reviewed above. Rather than serving merely as a post hoc interpretive lens, these theories guided the instructional architecture and interaction design of the system (Figure 1).

Specifically, Self-Determination Theory (SDT) informed the autonomy-supportive design of the AI environment. To promote learners' sense of agency and self-direction, the system was designed to encourage independent decision-making, reflection, and learner-generated responses rather than providing immediate answers. This design sought to support the psychological need for autonomy while fostering intrinsically motivated engagement.

Vygotsky's Zone of Proximal Development (ZPD) informed the adaptive scaffolding functions embedded within the AI Teaching Assistant. The system was designed to provide graduated support through prompts, hints, and guided questioning that responded to learners' evolving levels of understanding. Rather than replacing learner effort, support was intended to assist learners in accomplishing tasks that might otherwise remain beyond their current level of independent performance.

Socratic pedagogy informed the interactional logic of the AI Teaching Assistant. Instead of primarily delivering information, the system employed question-based guidance strategies that encouraged learners to justify their reasoning, elaborate their ideas, evaluate evidence, and engage in deeper cognitive processing. This questioning-oriented approach was intended to stimulate reflective thinking and active knowledge construction.

Finally, scaffolding-fading principles informed the temporal structure of the intervention. Across the 16-week learning period, instructional support was progressively reduced to encourage increasing learner responsibility and self-regulation. The design assumption was that learners would gradually rely less on external guidance and become more capable of independently initiating elaborative and metacognitive learning behaviors.

Together, these theoretical perspectives informed the architecture of the dual-role Agentic AI environment, the design of its instructional interactions, and the hypothesized pathways linking AI-supported learning processes to learner autonomy, self-regulated learning, and intrinsic motivation.

Uedu.tw is an open-access bilingual learning platform co-developed at National Central University, Taiwan. Its Agentic AI is built on a GPT-4-class LLM backbone with a pedagogically constrained prompting architecture and operates through two concurrently active roles. The AI Lecturer delivers structured English input, initiates Socratic questioning sequences, and adjusts task difficulty based on real-time performance modeling (task accuracy, response latency, learner-initiated help requests). The AI Teaching Assistant responds to learner queries via progressive hint scaffolding—reflection prompts, then conceptual cues, then worked examples only as a final step—with direct answer provision prevented by a Socratic guardrail at the prompt-architecture level. Scaffolding fading is algorithmic: hint specificity decreases as rolling weekly performance metrics improve (task accuracy ≥75%; declining self-initiated query rate); fading parameters were fixed throughout the intervention. Three additional capabilities relevant to participants' reported experiences (Section 3.4) operated continuously: a persistent learner-profile store enabling cross-session retrieval; automated post-session reports for extended practice tasks (e.g., simulated interviews); and supportive acknowledgment tokens triggered by engagement-signal features. The overall architecture is summarized in Figure 1.

#### 2.2.1 Intervention implementation

Weekly curriculum for both groups comprised four task types: (a) structured reading comprehension; (b) listening with comprehension prompts; (c) open-response short writing (120–200 words); and (d) a fourth task differing by condition—Socratic dialogue tasks (EG) vs. parallel open-response writing (CG). The intervention spanned 16 consecutive weeks. The control group used the identical Uedu.tw platform with Agentic AI functionality disabled, receiving the same curriculum and materials with static feedback on objective items and asynchronous instructor feedback on open-response tasks (typically within 48 h).

The principal between-group contrast therefore confounds AI access with task format (Socratic dialogue vs. parallel open-response writing); this co-variation is addressed in Section 4.4.

### 2.3 Measures

#### 2.3.1 Learner autonomy

The 24-item Learner Autonomy Scale for Language Learners (Lin and Reinders, 2017) was administered on a 5-point Likert scale across four dimensions (responsibility for learning, motivation/goal-setting, metacognitive awareness, independent learning behaviors); Cronbach's α = 0.89, McDonald's ω = 0.91.

#### 2.3.2 Self-regulated learning strategies

Three theoretically motivated MSLQ subscales (Pintrich et al., 1991): intrinsic goal orientation (4 items, α = 0.82), metacognitive self-regulation (12 items, α = 0.86), and elaboration (6 items, α = 0.80). Extrinsic goal orientation (4 items, α = 0.78) was retained as a theoretically informed discriminant comparison.

#### 2.3.3 Intrinsic L2 motivation

The 7-item Intrinsic Motivation subscale (α = 0.84) of the L2 Motivational Self System validated for East Asian EFL contexts (Taguchi et al., 2009). All instruments were administered in Traditional Chinese using forward–back translation (Brislin, 1970) with pilot testing (*n* = 15) prior to the main study.

#### 2.3.4 Platform interaction logs

Uedu.tw automatically logged all learner–AI interactions for EG participants (query text, system response type, timestamp, task context). Queries were classified as answer-seeking, elaboration-seeking, or metacognitive using an a priori coding scheme adapted from Aleven et al. (2003). Two trained coders independently classified 20% of queries (*n* = 1,247); intercoder agreement was strong (Cohen's κ = 0.84), with disagreements resolved through discussion before the remaining corpus was divided.

### 2.4 Semi-structured interviews

Sixteen EG participants were recruited through email invitation (first 16 respondents enrolled). Interviews were conducted in Mandarin Chinese, lasted approximately 25 min on average, and were audio-recorded with consent. Recordings were machine-transcribed and reviewed by the first author against the original audio; transcripts were engaged with in Mandarin to preserve nuance. The protocol covered four open-ended domains (overall AI-mediated learning experience, perceived behavioral changes, critical incidents, evaluation/future intentions) with flexible probing. We adopted integrative case summaries rather than verbatim quotation to preserve narrative coherence across conversational fragmentation; this is signaled by third-person reporting verbs and the absence of quotation marks.

### 2.5 Data analysis

#### 2.5.1 Quantitative analysis

Quantitative data were analyzed in SPSS 28 using one-way ANCOVA with pretest scores as covariates. ANCOVA assumptions (Shapiro–Wilk normality of residuals; homogeneity of regression slopes; Levene's test of error variance) were verified prior to analysis; no violations were detected. Effect sizes are reported as partial η^2^, consistent with the ANCOVA framework. The three primary outcomes—learner autonomy (LAS), the SRL strategy composite (MSLQ), and intrinsic L2 motivation (L2MSS)—are treated as distinct constructs; metacognitive self-regulation, a constituent subscale of the SRL composite, is reported as an exploratory within-composite subscale comparison rather than as an independent primary outcome. Alpha was set at 0.05; given three primary comparisons, a Holm–Bonferroni correction was applied. For the temporal analysis of AI Teaching Assistant queries, inference is conducted at the learner level: each participant's per-block proportion of elaboration-seeking and metacognitive queries was computed and the proportion showing a monotonic increase across the four time blocks is reported. The aggregate corpus-level chi-square test is reported descriptively only, given the nested data structure (queries within learners; see Section 4.4).

#### 2.5.2 Qualitative analysis and trustworthiness

Qualitative data were engaged in the spirit of Braun and Clarke (2019) reflexive thematic analysis, with an inductive–deductive hybrid orientation. The first author familiarized through repeated reading of all 16 transcripts in Mandarin; code–extract organization was maintained informally through annotations and memos rather than a formal coding matrix (a limitation noted in Section 4.4). Candidate themes were sharpened against sensitizing concepts from prior AI-mediated EFL work (Guo and Li, 2024; Ma and Chen, 2025; Wei, 2023) while preserving inductively emergent patterns. The first author is a platform co-developer, treated as both insight and potential bias; trustworthiness procedures comprised peer debriefing with the second author (C-KC, PI of the approved REC protocol) and member checking with six participants purposively selected across pretest LAS tertiles.

## 3 Results

### 3.1 Preliminary analyses

ANCOVA assumptions held: Shapiro–Wilk normality of residuals (*W* > 0.97, *p* > 0.05); Levene's test confirmed homogeneity of error variance (all *p* > 0.20); the homogeneity of regression slopes assumption held (group × pretest interactions all *p* > 0.15). Missing data <2%, pairwise deletion.

### 3.2 Effects on learner autonomy, SRL, and intrinsic motivation

ANCOVA results with pretest scores as covariates are summarized in Table 1. After Holm–Bonferroni correction, EG participants demonstrated greater posttest gains than CG peers across all primary outcomes. The between-group effect on the Learner Autonomy Scale was large, *F*(1, 117) = 38.42, *p* < 0.001, partial η^2^ = 0.247. The aggregate SRL strategy composite showed a comparable effect, *F*(1, 117) = 31.96, *p* < 0.001, partial η^2^ = 0.215. Intrinsic L2 motivation showed a large effect, *F*(1, 117) = 26.14, *p* < 0.001, partial η^2^ = 0.183. In an exploratory within-composite subscale comparison, metacognitive self-regulation—the largest constituent subscale of the SRL composite—showed a parallel effect, *F*(1, 117) = 33.01, *p* < 0.001, partial η^2^ = 0.220; because this subscale is a component of the composite rather than an independent construct, it is reported as an exploratory comparison and is not included in the family-wise correction. Consistent with the discriminant-comparison prediction derived from SDT, the extrinsic goal orientation subscale did not differ significantly between groups, *F*(1, 117) = 1.58, *p* = 0.21, partial η^2^ = 0.013. These effects are large (Cohen, 1988); the intrinsic–extrinsic dissociation is consistent with an SDT-based interpretation, with magnitude developed in Section 4.

**Table 1:** Adjusted means and ANCOVA results for primary and exploratory outcomes (*n* = 60 per group).

Measure	EG Adj. *M*	CG Adj. *M*	*F*(1,117)	*p*	partial η^2^
Primary Outcomes	
LAS total	3.80	3.19	38.42	<0.001	0.247
SRL (MSLQ composite)	3.85	3.30	31.96	<0.001	0.215
Intrinsic motivation	3.73	3.23	26.14	<0.001	0.183
Exploratory Subscale Comparisions	
Metacognitive regulation	3.68	3.12	33.01	<0.001	0.220
Extrinsic goal orientation	3.27	3.25	1.58	0.210	0.013

Adjusted means controlling for pretest scores. Effect sizes are reported as partial η^2^, consistent with the ANCOVA framework. Metacognitive Regulation is presented as an exploratory within-composite subscale comparison. After Holm–Bonferroni correction across the three primary outcomes, EG participants demonstrated greater posttest gains than CG peers on LAS, SRL composite, and intrinsic motivation; exploratory analyses indicated a substantial between-group difference on metacognitive regulation, whereas extrinsic goal orientation did not differ significantly.

### 3.3 Developmental shift in AI query patterns

Across the 16-week intervention, EG participants generated 6,235 AI Teaching Assistant queries. We frame the developmental analysis at the learner level: of the 60 EG participants, 54 (90.0%) showed a monotonic increase across the 4-week blocks in the combined proportion of elaboration-seeking and metacognitive queries, indicating that this query-pattern shift was distributed across the cohort rather than driven by a small number of high-engagement users. We report this as a descriptive behavioral pattern. Because task type and difficulty were not held constant across the four blocks and self-regulation was not independently measured at each block, the shift is open to non-developmental explanations—including growing familiarity with the interface, repeated exposure to the system's Socratic response style, and changing task demands—and should not be read as direct evidence of internalized self-regulation (see Section 4.2).

For descriptive corpus-level reference, the aggregate query distribution shifted from 71.3% answer-seeking and 7.3% metacognitive in Weeks 1–4 to 28.9% answer-seeking and 36.9% metacognitive by Weeks 13–16; because queries are nested within learners, this aggregate pattern is reported descriptively, with the learner-level monotonicity statistic above serving as the principal evidence.

### 3.4 Qualitative findings

Reflexive thematic analysis of the 16 interviews yielded three themes, illustrated through integrative case summaries—synthesized third-person descriptions drawn from multiple points across each interview. Following Braun and Clarke (2019), we describe relative theme prominence using qualitative descriptors. Participants are identified by anonymised codes; caveats on the qualitative evidentiary basis (EG-only sample, co-developer analyst, integrative case summaries rather than verbatim quotation) are discussed in Sections 2.5.2 and 4.4(5).

***Theme 1. Perceived Agency Through Self-Paced Interaction (most prominent theme)***

The most prominent theme concerned a reconfiguration from externally driven obligation toward self-paced practice embedded in daily life. P01 described a shift from experiencing English as a “task to be completed” toward autonomous engagement, developing a daily commute-time practice habit; P05 reported enhanced self-regulation through the system's proactive acknowledgments; P16 described the platform as his “daily English chatroom,” attributing sustained engagement to the visualization of weekly progress. Across these cases, self-paced adaptive difficulty, daily-habit formation, and visible progression tracking appeared as mutually reinforcing mechanisms of perceived agency.

***Theme 2. Adaptive Feedback as ZPD Scaffolding (secondary theme)***

A second theme concerned the calibrated character of the AI's feedback, mapping closely onto the ZPD principle of scaffolding pitched just beyond independent capacity. P02 described a shift from passive receipt to active dialogic engagement through Socratic follow-up questions; P03 contrasted traditional weekly teacher feedback with sentence-level immediacy that increased her willingness to attempt unfamiliar constructions; P11 described cross-session contextual memory linking weekly practice to her stated travel plan; P12 described a structured post-session report identifying response-timing patterns. Across these cases, learners described feedback with three distinctive properties: timing immediacy at the level of individual utterance, contextual specificity drawing on prior interactional history, and a dual-function quality combining correction with forward-directed progression.

***Theme 3. Affective Safety and Relational Warmth (supplementary theme)***

A third theme, less prominent but emergent across several participants, described a dimension not anticipated by the initial SDT–ZPD framework: the AI as an affectively safe and relationally warm partner. P04 reframed the AI's non-human status as enabling linguistic experimentation free of peer evaluation; P10 was struck by the system treating his affective state, not merely his linguistic output, as a legitimate pedagogical object; P13 felt English learning connected to self-expression rather than performance. P15 offered the most qualified endorsement, positioning the AI as a “practice partner” rather than “replacement” and noting that human warmth and accent diversity remain beyond AI's current range. These cases surface an affective-relational dimension developed further in Section 4.3.

## 4 Discussion

This study examined whether a Socratic, dual-role Agentic AI may support the development of learner autonomy, self-regulated learning (SRL), and intrinsic motivation in an EFL higher education context. Three findings warrant discussion: convergent quantitative effects, the developmental shift in learner–AI interaction, and the interpretive limits of the present design—particularly the distinction between within-intervention performance and durable autonomy transfer.

### 4.1 Convergent evidence and theoretical coherence

The between-group effect on the Learner Autonomy Scale was large (Table 1), comparable to or exceeding effects reported in prior technology-enhanced autonomy interventions (Zheng et al., 2018). More important than magnitude is theoretical coherence: gains on intrinsic goal orientation and metacognitive self-regulation, coupled with the absence of effect on extrinsic goal orientation, align with the dissociation predicted by SDT's distinction between autonomous and controlled motivational regulation (Deci and Ryan, 2000). This pattern is difficult to attribute to generalized novelty or expectancy effects, which would inflate all motivational measures uniformly; it is more consistent with the AI-supported condition engaging the psychological mechanisms SDT identifies as foundational to autonomy internalization (Ma and Chen, 2025; Wei, 2023).

These findings converge with prior work documenting motivational gains in AI-mediated language learning (Fathi et al., 2024; Ma and Chen, 2025; Wei, 2023) and extend it by tracing a within-intervention behavioral trajectory that complements the outcome-level designs of Guo and Li (2024) and Ebadi and Amini (2024). The AI-presence and task-format components of the between-group contrast are addressed in Sections 4.3 and 4.4.

### 4.2 A descriptive shift in learner–AI query patterns

The developmental shift in AI Teaching Assistant query patterns—with 90.0% of EG participants exhibiting a monotonic learner-level shift toward elaboration-seeking and metacognitive queries—is descriptively consistent with sociocultural and SRL accounts in which co-regulated interaction is progressively internalized (Lantolf and Thorne, 2006; Zimmerman, 2000). We are careful, however, not to overinterpret this pattern. The monotonicity statistic does not by itself establish internalization or durable self-regulation: alternative explanations—growing platform familiarity, exposure to the Socratic response style, and unequal task type and difficulty across blocks—cannot be ruled out with the present data. Establishing internalized self-regulation would require designs that disentangle task progression from learner change and measure self-regulation outside the system.

### 4.3 The autonomy–accessibility paradox

We turn to the central interpretive caveat. The favorable outcomes documented above describe learners' behavior and self-report while embedded within the AI-scaffolded environment; whether these gains translate into durable autonomy once support is removed is a question the present concurrent design cannot resolve. This reflects Salomon et al. (1991) distinction between effects with technology (performance enabled while in use) and effects of technology (cognitive residue persisting after removal). We name this concern the autonomy–accessibility paradox: the design features that render AI support pedagogically effective—immediate availability, adaptive calibration, frictionless engagement—may simultaneously attenuate the productive cognitive struggle through which self-regulation is internalized (Darvishi et al., 2024; Kirschner and van Merriënboer, 2013). Resolving this requires longitudinal designs that directly test transfer after AI withdrawal.

A second interpretive move concerns the affective-relational dimension surfaced by Theme 3, which points toward a complementary strand of inquiry on affective computing and care ethics in AI-mediated learning—a generative direction for future theoretical development rather than a conclusion warranted by the present data.

Three implications follow: models of AI-mediated learner autonomy should incorporate the temporal dimension of scaffolding withdrawal; future research should incorporate delayed unscaffolded post-tests; and the field would benefit from graduated AI withdrawal protocols as a pedagogical complement to within-session scaffolding fading.

### 4.4 Limitations

Five limitation themes qualify the interpretation of these findings.

1. Design-level confounds. The quasi-experimental design with intact-class allocation introduces potential class-level clustering that, with only two clusters, multilevel modeling cannot address (Maas and Hox, 2005); identical instructor and curriculum, and verification of baseline equivalence, partially mitigate this concern. Additionally, the principal between-group contrast comprises two co-varying components—AI access and Socratic-dialogue versus open-response task format—and observed effects cannot be cleanly attributed to AI-system functionality alone. Future designs holding task format constant while varying AI scaffolding would help disentangle these components.
2. Concurrent assessment cannot diagnose autonomy transfer; longitudinal follow-up with delayed unscaffolded post-tests is the most pressing design need (Section 4.3).
3. Pseudoreplication. The 6,235 queries are nested within 60 learners across four blocks; inference is framed at the learner level (90.0% monotonic shift), with the aggregate chi-square descriptive only. A formal multilevel approach (e.g., generalized linear mixed models with learner-level random effects) is a priority for follow-up reporting.
4. Sample, power, and engagement metrics. The achieved sample (*N* = 120) fell short of the a priori target (*N* = 128); effect sizes are interpreted with appropriate caution regarding precision rather than via post hoc power. We did not include a between-condition statistical comparison of platform engagement metrics (session frequency and duration), so cannot empirically rule out differential engagement as a partial contributor; we identify this as a priority for follow-up reporting. Novelty effects are mitigated by the 16-week horizon and the intrinsic–extrinsic dissociation; teacher effects by the single-instructor design.
5. Qualitative formality, researcher positioning, and generalisability. The qualitative component is reported at a level appropriate to a Brief Research Report but short of a fully systematic reflexive thematic analysis (no formal code–extract matrix, continuously maintained reflexive journal, or audit trail). Three further constraints warrant a modest reading: interviews were conducted with experimental-group participants only, so themes cannot establish AI-condition distinctiveness; participants were the first 16 volunteers, with probable self-selection toward more favorably disposed learners; and the analysis was led by a platform co-developer despite the trustworthiness procedures described in Section 2.5.2. Independent replication with control-group interviews and an analyst external to the platform is warranted. The single-institution sample also constrains generalisability across CEFR proficiency levels and institutional types, and the LLM backbone reflects a specific technology generation, although the theoretical mechanisms examined (Socratic guardrails, scaffolding fading) are architecture-agnostic.

### 4.5 Conclusion

Under the conditions examined here, a dual-role Agentic AI with Socratic guardrails and algorithmic scaffolding fading was associated with theoretically coherent gains in learner autonomy, SRL, and intrinsic L2 motivation among Taiwanese college EFL learners, alongside a descriptive learner-level shift from answer-seeking toward metacognitive query patterns. These associations must be interpreted against three caveats: the between-group contrast confounds AI presence with task format; the query-pattern shift is a descriptive within-system trajectory rather than established internalization; and the autonomy–accessibility paradox renders unresolved whether within-intervention gains persist after AI withdrawal. Advancing the field will require designs that disentangle AI scaffolding from task format and treat scaffolding withdrawal as a first-class pedagogical problem.

## Data Availability

The raw data supporting the conclusions of this article will be made available by the authors, without undue reservation.
